# Oxidative Stress and DNA Damage Effect of *Dioscorea hispida* Dennst. on Placental Tissues of Rats

**DOI:** 10.3390/molecules27072190

**Published:** 2022-03-28

**Authors:** Hussin Muhammad, Tengku Aideed Syah Tg Abu Bakar, Muhamad Faizul Adhzim Yusery, Norizah Awang, Wan Mazlina Md. Saad, Elda Nurafnie Ibnu Rasid, Mohamad Fawzi Mahomoodally, Maizatul Hasyima Omar

**Affiliations:** 1 Herbal Medicine Research Centre, Institute for Medical Research, National Institutes of Health, Jalan Setia Murni U13/52, Seksyen U13, Setia Alam, Shah Alam 40170, Selangor, Malaysia; norizah@moh.gov.my (N.A.); maizatul.hasyima@moh.gov.my (M.H.O.); 2 Department of Medical Laboratory Technology, Faculty of Health Sciences, Universiti Teknologi MARA UiTM Puncak Alam, Bandar Puncak Alam 42300, Selangor, Malaysia; midham@uitm.edu.my (T.A.S.T.A.B.); faizul_adhzim@yahoo.com (M.F.A.Y.); wanmaz755@salam.uitm.edu.my (W.M.M.S.); 3 Department of Health Sciences, Faculty of Medicine and Health Science, University of Mauritius, Réduit 80837, Mauritius; f.mahomoodally@uom.ac.mu

**Keywords:** *Dioscorea hispida*, comet assay, oxidative stress, reactive oxygen species, placental tissue

## Abstract

*Dioscorea hispida* Dennst. locally known as “ubi gadung” has been used as a traditional remedy and source of carbohydrate among Malaysians. To assess the effect of *Dioscorea hispida* aqueous extract (DHAE) on the production of reactive oxygen species (ROS) and their effects on DNA damage in Sprague Dawley rat’s placental tissues, pregnant rats were randomly divided into four groups. The animals were orally treated with distilled water (negative control) and three different concentrations of DHAE (250, 500 and 1000 mg/kg body weight (BW)) from gestation day 6 until 20. The oxidative stress in placental tissues was evaluated at day 21 by measuring the level of ROS, superoxide dismutase (SOD) and lipid peroxidation biomarker, malondialdehyde (MDA) while comet assay was used for DNA damage. There was no significant production of ROS and SOD activities in all groups. Significant changes were observed in the MDA level at 1000 mg/kg BW DHAE. Comet assay revealed a significant increase (*p* < 0.05) of DNA damage on animals treated with 250 and 500 mg/kg BW DHAE but not at the highest concentration. It was postulated that the placental cells could have undergone necrosis which destroys all components including DNA. This occurrence simultaneously reduces the levels of DNA damage which can be represented by lower level of tail moments. This finding correlates with our histopathological examination where necrotic cells of spongiotrophoblast were observed in the basal zone of placental tissue. The high amount of hydrogen cyanide and other compounds in 1000 mg/kg BW DHAE could elevate the lipid peroxidation and directly induce cell necrosis which requires further investigation.

## 1. Introduction

Normal embryonic/fetal development requires a specific signaling mechanism that regulates cell proliferation and differentiation. It is known that abundant reactive oxygen species (ROS), which is the by-product of cellular metabolic activity, is produced during organogenesis [1]. The most common ROS are superoxides anion (O^2−^), hydrogen peroxides (H_2_O_2_) and hydroxyl radical (OH^−^) [2]. During pregnancy, the placenta will undergo appropriate development for fetal growth and development. Any intake of xenobiotics such as drugs or herbal products may cause chemical compounds transmission through the placenta and produce an excessive amount of ROS which lead to the disruption of cellular signaling in fetal growth and results in adverse outcomes such as preterm birth [3], intrauterine growth retardation [4], congenital defects [5] and low birth weight [6].

*Dioscorea* or commonly known as yam is the edible starchy plant from the family of *Dioscoreaceae*. The genus of *Dioscorea* consists of about 630 species and is distributed around the world but the major cultivated species are *D. alata*, *D. esculenta*, *D. pentaphylla*, *D. pubera*, *D. villosa*, *D. acuelata*, *D. orbiculata* and *D. hispida* [7]. In Malaysia, *D. hispida* is called “ubi gadung” and is typically found in the eastern region. It is classified as a wild creeping plant that grows up to 20 m. The tuber is lobed with the presence of starch granules in variable sizes and covered with dead roots [8].

In Malaysia and Indonesia, *D. hispida* is traditionally consumed as one of the main sources of carbohydrates and it was detoxified by soaking it under flowing water for a week prior to consumption [9]. Steroidal saponins from yam tubers were found to have a wide range of pharmacological activities such as anthelmintic, lowering the cholesterol levels, antioxidant, analgesic, anti-inflammatory and anti-tumor (Nasyirah et al., 2014). However, this species also contains an alkaloid known as dioscin and a high concentration of cyanogenic glycosides [9] which potentially produce toxic effects on fetal development.

Although *D. hispida* has become a staple food for certain parts of the countries, the effect of its consumption on pregnant women was not elucidated. Therefore, the present study was conducted to investigate the effects of *D. hispida* aqueous extract on the production of ROS and DNA damage in placental tissue in pregnant rats during organogenesis.

## 2. Results

### 2.1. UHPLC-ESI-MS Analysis

**Figure 1:**
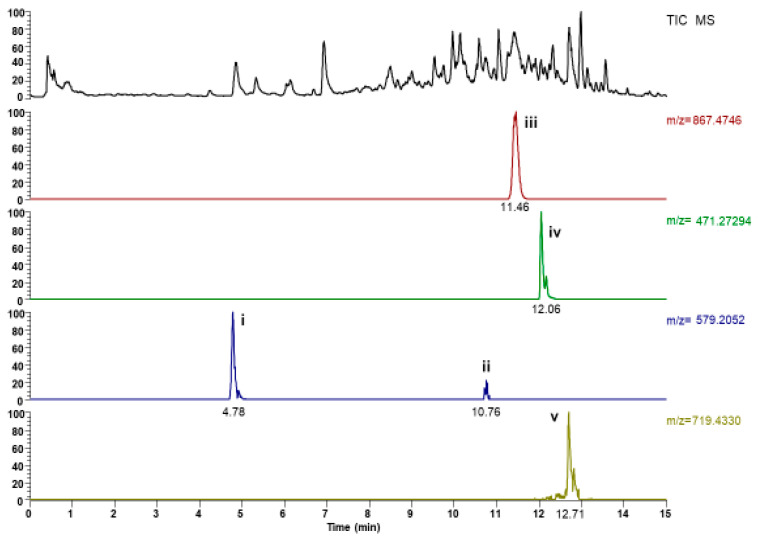
Chromatograms of the extracts from *D. hispida* analyzed by UHPLC. (i and ii) (+)syringavesinol-4-*O*-β-d-glucopyranoside-isomer (iii) Dioscin (iv) Diosgenin-3-6-dione (v) Progenin III.

In the present study, UHPLC-ESI-MS has been applied to characterize the steroidal saponins in *D. hispida*. A total of four steroidal saponins were detected in which they were tentatively identified based on retention time, accurate molecular ion (*m*/*z*), molecular formula with reference to the reference standard and data from the previous study (Table 1). The chromatogram of aqueous extract from the tubers of *D. hispida* is shown in Figure 1.

Two peaks (peaks i and ii), having the same formula but different retention time, were detected and characterized to be two isomers of (+)Syringavesinol-4-*O*-β-d-glucopyranoside. Peak iii with a retention time of 11.46 min produced a strong deprotonated molecule [M − H]^−^ (*m*/*z* = 867.47941) in (−)-ESI-MS that was tentatively identified as dioscin (Zhu et al., 2010). Peak iv (Rt = 12.0 min) showing deprotonated molecular ion [M − H]^−^ (*m*/*z* = 471.2748) was tentatively identified as diosgenin-3,6-dione. Peak v (Rt = 12.71 min) readily produced a strong deprotonated molecular ion at [M − H]^−^ (*m*/*z* = 719.4370) in (−)ESI-MS. Considering the molecular mass, peak v was identified as Progenin III, which has been identified previously from D.cayenensis [10]. D. hispida aqueous extract yielded 243 ± 2.1 mg gallic acid equivalent (GAE)/100 g of total phenolic content.

### 2.2. Determination of ROS Level

**Figure 2:**
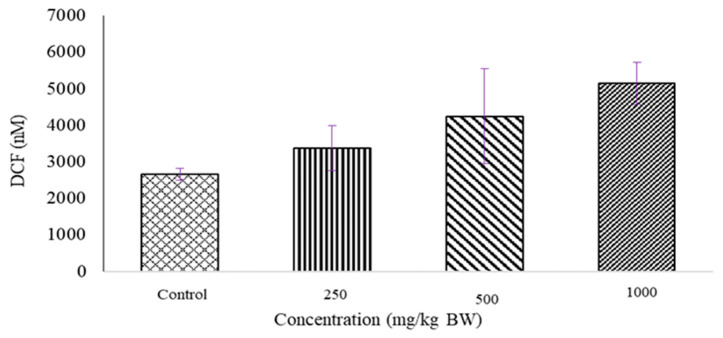
Production values of ROS in placenta tissues of pregnant rats treated with *D. hispida* aqueous extract at 250, 500 or 1000 mg/kg BW body weight for 15 days. Values of ROS based on the intensity of 2′,7′-Dichlorodihydrofluorescein (DCF) were expressed as mean ± SEM (*n* = 5) (*p* > 0.05).

A Reactive Oxygen Species (ROS) production assay was conducted to assess oxidative stress induction in the placental tissues of pregnant rats. Figure 2 shows the level of ROS production in placental tissues after 15 days of administration of *D. hispida* aqueous extract. Dose dependent in ascending order of ROS which translated in DCF value was observed in all placenta tissue (Control, 250, 500 and 1000 mg/kg BW: 2657.7 ± 160.36 nM, 3379.6 ± 616.41 nM, 4246.6 ± 1303.2 nM, 5153.7 ± 578.52 nM) though the increment was not significant).

### 2.3. Lipid Peroxidation and Malondialdehyde (MDA) Quantification Assays

**Figure 3:**
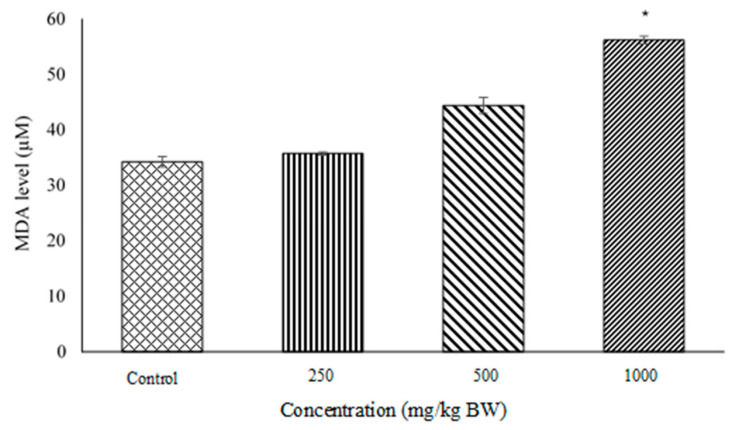
MDA level (µM) in pregnant rat’s placenta. The bar chart shows the level of lipid peroxidation by-product in the maternal rat’s placenta of control, 250, 500 and 1000 mg/kg BW. Data values were expressed with mean ± SEM (*n* = 5). * Significant difference between control and 1000 mg/kg BW (*p* = 0.05).

MDA level in pregnant rat’s placental tissues (Figure 3) is proportional to the lipid peroxidation process. Rats treated with *D. hispida* extract showed significant difference in DNA level between Control vs. 1000 mg/kg BW (34.231 ± 0.966 µM; 56.191 ± 0.729 µM), and 250 mg/kg (35.732 ± 0.293 µM) vs. 1000 mg/kg BW.

### 2.4. Superoxide Dismutase (SOD) Activity Assay

**Figure 4:**
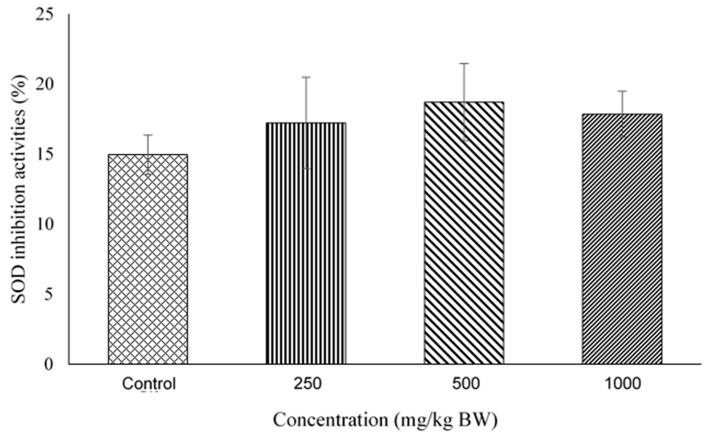
SOD inhibition activities in maternal rat’s placenta. The bar chart shows the percentage of SOD inhibition activities in the maternal rat’s placenta of control, 250, 500 and 1000 mg/kg BW. Data values were expressed with mean ± SEM (*n* = 5). No significant difference between all groups (*p* > 0.05).

Figure 4 shows the SOD inhibition activities in pregnant rat’s placenta. The SOD inhibition values in percentage for 250, 500 and 1000 mg/kg BW were 17.225 ± 3.259, 18.699 ± 2.755, 17.841 ± 1.639 respectively, whilst in control was 14.957 ± 1.395. No significant differences were observed between these groups.

### 2.5. Determination of DNA Damage Using Comet Assay

**Figure 5:**
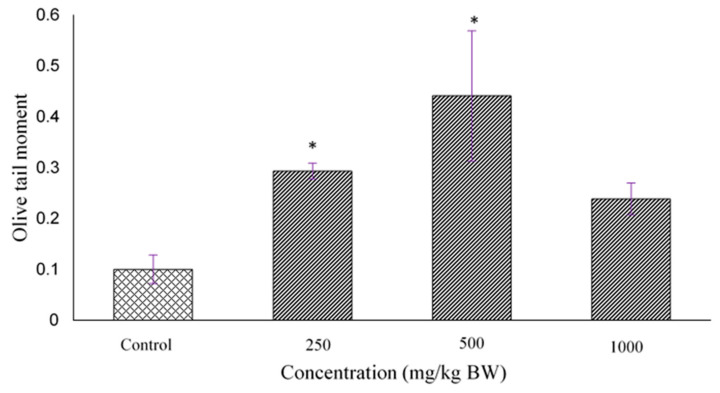
The chart above shows the olive moment values of DNA damage. The pregnant rats were treated with *D. hispida* aqueous extract at 250, 500 and 1000 mg/kg BW for 15 days. Tissues were subjected to alkaline comet assay and visualized under confocal laser microscope after staining with vista green. All the data were analyzed for tail DNA percentage and tail moment length by OpenComet v1.3.1 using ImageJ version 1.53. Values were expressed as mean ± SEM (*n* = 5). * Indicates significant difference compared with control (*p* < 0.05).

**Figure 6:**
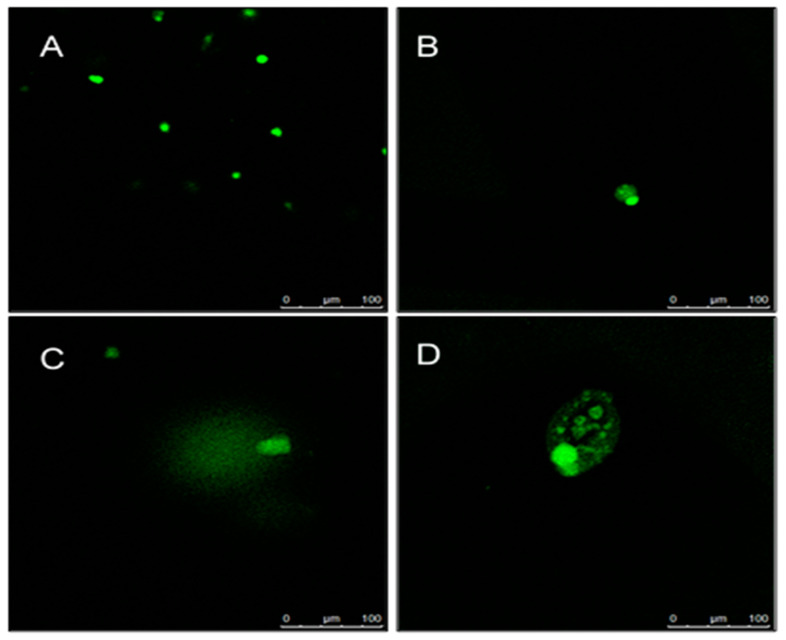
Evaluation of genotoxic effects induced by different concentrations of *D. hispida* aqueous extract. (**A**) control: nucleus without DNA damage (**B**) 250 mg/kg BW: mild DNA damage (**C**) 500 mg/kg BW: moderate DNA damage (**D**) 1000 mg/kg BW: nucleus with necrotic cell. The final data were presented as an olive tail moment (OTM).

DNA damage in rat’s placenta tissues presented as olive tail moment (Figure 5) and the photographs of the comet are shown in Figure 6. The value of olive tail moment for control (0.1 ± 0.0161) 250 (0.29 ± 0.0893), 500 (0.44 ± 0.074), 1000 mg/kg BW (0.24 ± 0.018). The olive tail moment in 250 and 500 mg/kg were significantly different than in control, with *p* < 0.05 respectively.

## 3. Discussion

The use of herbal plants as a source of medicine and food has become a common practice among pregnant women due to easy access and belief that they are safe for consumption [11]. The biological activity of these plants is contributed to by the presence of abundant amounts of compounds and some of them may be potentially harmful and cause toxicity especially if taken during pregnancy. 

The potential toxicity effects of administration of *D. hispida* aqueous extract on the ROS generation and DNA damage in placental tissue of pregnant rats during the organogenesis period were studied. Our findings demonstrated that no mortality, behavioral and other toxicological clinical changes were recorded in dams treated with *D. hispida* extract up to 1000 mg/kg. Similar observations have been reported earlier by Xu et al. [12] in non-pregnant rats treated with repeated doses of 300 mg/kg of *D. hispida* extract for 90 consecutive days. In treatment with other species of *Dioscorea*, administration of *D. zingiberensis* at 255 mg/kg for 30 days has no significant impact on its biochemical and hematological parameters changes [13].

The placenta plays an important role to support the normal growth and development of the fetus during pregnancy. The function of the placenta is to provide oxygen and nutrients and to protect the fetus against xenobiotic compounds, infections and maternal diseases. Therefore, any disturbances to this function will affect the normal functional activity of the placenta to accommodate the high demand of developing fetuses. Preclinical studies in vivo and in vitro studies have demonstrated the embryotoxicity potential of some of the herbal plants and their formulations [14]. Our previous results confirmed that *D. hispida* extract has no effect on the placenta weight and external morphological changes in fetuses. However, the histological examinations of the placenta showed varying degrees of basal and labyrinth zone damage and cellular disruption at 250, 500 and 1000 mg/kg body weight *D. hispida* aqueous extract [15]. To understand the mechanism involved in the later effects, we investigated the cellular activity of the extract and whether it may act as an exogenous stimulus in reactive oxygen species (ROS) production and cause placental DNA damage. 

The source production of ROS is characterized as endogenous and exogenous where the endogenous sources are the mitochondria, plasma membrane, endoplasmic reticulum and peroxisomes [16]. During normal pregnancy, the placenta circulation and high metabolic demands of the fetus become an additional source of ROS generation that contributes to oxidative stress. As previously reported, during normal pregnancy, placental cells secrete SOD as an adaptive defense mechanism to counteract the raised oxidants and reduce the oxidative stress [17]. Our data revealed that treatment with *D. hispida* extract increased ROS levels and SOD activity in the placenta of rats in a dose dependent manner, but was not significantly different when compared to control. The excessive accumulation of ROS will trigger DNA damage through the interruption of mitochondria. The potential effect of *D. hispida* aqueous extract to cause DNA damage was assessed using single cell electrophoresis assay, well known as comet assay. The present study showed a significant level of DNA damage in treated (250 and 500 mg/kg body weight) groups as compared to control. However, a similar effect was not observed in the placental tissue from the 1000 mg/kg body weight *D. hipida* group as the cells could have undergone necrosis which destroys all components including DNA, hence the assay was unable to detect the DNA damage. This is evident in our histopathological examination of placental tissue where necrotic cells of spongiotrophoblast were observed in the basal zone [15]. This occurrence simultaneously reduces the level of DNA damage which can be represented by lower levels of tail moments [18]. 

Lipid peroxidation is a process in which free radicals or non-radical species attack polyunsaturated fatty acids (PUFAs) and cause direct damage to the lipid pathway. The secondary by-products of lipid peroxidation are lipid hydroperoxide, malondialdehyde (MDA), propanol, hexanal and 4-hydroxynonenal [19]. MDA appears to be the most mutagenic product, and hence it has been used as a biomarker for lipid peroxidation [20]. The metabolism of MDA will further generate malondialdehyde acetaldehyde (MMA) adducts resulting in DNA damage or cell death which potentially interfere with placental and fetal development [21]. In our study, the level of MDA was increased in ascending order with the administration of *D. hispida* aqueous extract. These findings suggest that the administration of the extract at the highest concentration (1000 mg/kg body weight) could cause the loss of cellular function of the placental membrane due to lipid peroxidation and promote cell necrosis as we observed in the comet assay. 

Phytochemical analysis of *D*. *hispida* aqueous extract via LCMS revealed the presence of several steroidal saponins such as dioscin and progenin III which are similar to the previous findings [10,22]. Dioscin has been suggested to have potent effects against liver fibroblast, renal injury and cerebral ischemia injury. It has also indicated that dioscin can prevent mitochondrial apoptosis and attenuated oxidative stress in cardiac cells [23]. Dioscin significantly decreased the levels of MDA, SOD, NO and iNOS in mice and rats [24]. Another study by Zhao et al. [25] also demonstrated that dioscin markedly decreased the ROS and MDA in H9C2 cells (doxorubicin-induced cardiotoxicity) [25]. However, these biological findings were contradicted with our results. It is known that different extractions could result in diverse chemical compounds and pharmacological activities. Therefore, it is postulated that *D. hispida* might consist of other compounds such as cyanogenic glycosides that contribute to its toxicity effects. Cyanogenic glycosides i.e., diosgenin are natural toxins found in food plants including bitter toxic species of *Discorea* such as *D. dumetorum*, *D. hirsuta*., and *D. bulbifera* [16]. One of the common toxic chemical compounds found in discorea spp, cyanide increases the production of ROS when it is converted into hydrogen cyanide (HCN) through the hydrolysis process resulting in DNA damage [26]. The high amount of these compounds in 1000 mg/kg body weight of *D. hispida* aqueous extract could potentially elevate the lipid peroxidation and directly induce cell necrosis, hence requiring further studies.

## 4. Materials and Methods

### 4.1. Chemicals

Folin-Ciocalteau (Sigma Aldrich, St. Louis, MO, USA), Formic acid (Merck, Darmstadt, Germany), acetonitrile (Merck), OxiSelect™ Intracellular ROS Assay Kit (Green Fluorescence), OxiSelect™ TBARS Assay Kit (MDA Quantitation) and OxiSelect™ Superoxide Dismutase Activity Assay Kit (Cell Biolabs Inc., San Diego, CA, USA) and diethyl ether (Merck).

### 4.2. Plant Material and Extract Preparation

*D. hispida* fresh tubers were collected from Machang, Kelantan. The plant was authenticated by Forest Research Institute Malaysia (FRIM) with the voucher specimen labeled (SBID:0012/08). The tubers were washed, dried, and ground to powder form. The powder (250 mg) was then extracted with hot water (80 °C) and filtered. The filtrate was collected and dried with a spray dried machine (Bücchi Labortechnik AG, Flawil, Switzerland). The extract obtained was diluted with water to 10 mg/mL and filtered through a 0.22 µm PTFE membrane prior to Ultra High-Performance Liquid Chromatography ElectroSpray Ionization Mass Spectrometry (UHPLC-ESI-MS) analysis.

### 4.3. Total Polyphenols

The total polyphenols assay was performed using a Folin-Ciocalteau assay based on a method of Singleton and Rossi (1965) [27]. The method allows the determination of oxidised phenols by producing a blue colour from heteropoly phosphomolybdate-tungsten anions where the darker the blue colour the more phenols are present.

### 4.4. UHPLC-ESI-MS Analysis

The liquid chromatographic system was a QExactive UHPLC (Thermo Fisher Scientific, Waltham, MA, USA) composed of the following components: binary pump, a solvent degasser, an autosampler, and a thermostatically controlled column compartment. Separation was achieved on WATERS C18 (2.1 × 50 mm, 17 µm) column (Waters, Milford, MA, USA). The mobile phase consisted of water with 0.1% formic acid (A) and acetonitrile with 0.1% formic acid (B) at a flow rate of 0.4 mL/min. The following solvent composition was used: 0–5 min, 10–20% B (linear gradient); 5–10 min, 20–60% B (linear gradient); 10–13 min, 60–90% B (linear gradient); 14–14.10 min, 90–10% B (linear gradient) and 14.1–15 min, 10% B (isocratic).

### 4.5. Animals and Experimental Design

Eight weeks-old virgin female (*n* = 20) and fertile male (SD) (*n* = 10) Sprague-Dawley rats weighing from 180–250 g were supplied by the Laboratory Animal Resource Unit (Medical Research Resource Centre, Institute for Medical Research, Malaysia). Animals were housed in rat standard polypropylene cages under controlled temperature (20 ± 2 °C) and humidity (40–60%) with a 12 h light–dark cycle, and given ad libitum access to food (Specialty Feeds, Glen Forrest, Australia) and water. All rats were acclimatized for 7 days prior to the start of the study. Approval for this study was obtained from the Animal Care and Use Committee, Ministry of Health Malaysia (ACUC No: ACUC/KKM/02(10/2016).

Vaginal smears were performed to determine the estrous cycle. Female rats in the pro-estrous phase were placed into the cage of male in the late morning (1:1) and left for 24 h. The presence of sperm in the vaginal smear was considered as gestation day 0 (GD 0). The animals were treated with distilled water (negative control) and three different concentrations of *D. hispida* aqueous extract (DHAE). DHAE was dissolved in distilled water to formulate three concentrations (250, 500 and 1000 mg/kg body weight). The extracts were freshly prepared and administered orally using an intubation needle (volume 10 mL/kg) to pregnant rats daily from gestation day 6 until 20. On day 21, rats were anesthetized using diethyl ether and caesarean section was performed. The gravid uterus was removed and placentas were collected and immediately stored at −80 °C prior to analysis.

### 4.6. Determination of ROS Level

The placental tissues were soaked in 1X phosphate buffered saline (PBS) containing ethylenediaminetetraacetic acid (EDTA). For each sample, the placenta tissue was homogenized by mortar and pestle on ice and mixed with 1 mL of PBS. The tissues were then centrifuged at 10,000 rpm, at 4 °C for 10 min and the supernatant was collected. It was repeated for the next groups of samples. The supernatant of an unknown sample (approximately 50 µL) or hydrogen peroxide (H_2_O_2_) standard was then collected and added into a 96-well plate, followed by the addition of 50 µL of catalyst in each well and incubation for 5 min at room temperature. A total of 100 µL of dichlorodihydrofluorescin (DCFH) solution was added into each well. The microplate was incubated at room temperature for 45 min. The fluorescence was read at 485 nm excitation/520 nm.

### 4.7. Lipid Peroxidation and Malondialdehyde (MDA) Quantification Assays

The MDA level in rat’s placenta tissue was determined using OxiSelect™ TBARS Assay Kit (MDA Quantitation). Blood was removed from tissue samples by perfusion with PBS containing EDTA. Tissues were re-suspended at 50 to 100 mg/mL in PBS containing 1X Butylated Hydroxytoluene (BHT) and homogenized on ice, then centrifuged at 10,000× *g* for 5 min to collect the supernatant. MDA in samples and standards were reacted and incubated with Thiobarbituric Acid (TBA) at 95 °C for 45–60 min. The MDA standards and samples were transferred to a 96 well microplate compatible with a spectrophotometric plate reader and read at 532 nm. The MDA level in the sample was determined by the MDA standard curve.

### 4.8. Superoxide Dismutase (SOD) Activity Assay

The SOD activity in rat’s placenta tissue was determined by OxiSelect™ Superoxide Dismutase Activity Assay Kit. Tissue samples were homogenized with 5–10 mL of cold 1X lysis buffer and centrifuged at 12,000× *g* for 10 min. The supernatant was collected for further analysis. Xanthine/Xanthine Oxidase reagent was reacted with the supernatant to generate superoxide anions which were detected by chromogen solutions and the absorbance was read at 490 nm with a microplate reader (POLARstar Omega, BMG Labtech, Ortenberg, Germany). The activity of SOD was determined as the inhibition percentage of chromogen reduction.

### 4.9. Determination of DNA Damage Using Comet Assay

The DNA damage was quantified by measuring the displacement between the genetic material of the nucleus (‘comet head’) and the resulting ‘tail’. Tail Moment and Tail DNA% are the two most common parameters for Comet assay. Fifty cells were scored and analyzed per sample. The Olive Tail Moment was calculated and automatically analysed by using Open Comet 1.3 using Image J software. The Olive Tail Moment was calculated as follows: Olive Tail Moment = Tail length × % of DNA in the tail.

### 4.10. Statistical Analysis

The data were analyzed with SPSS version 21.0 (IBM, Armonk, NY, USA) by utilizing analysis of variance (ANOVA) and followed by Tukey post-hoc test. The values of Comet assay are shown as mean ± SEM and were analyzed using the Kruskal Willis test. The results were considered as significant when (*p* < 0.05).

## 5. Conclusions

These findings suggest that oral administration of *D. hispida* aqueous extract in pregnant rats potentially induces the elevation of ROS production, lipid peroxidation and DNA damage through necrosis to the placental tissue at 1000 mg/kg BW equivalent to human concentration 162.16 mg/kg BW. Further research should be carried out to elucidate the proinflammatory cytokines markers such as IL-1, IL-2, IL-8, TNF-α and IFN-γ which are known to be associated with several pregnancy complications. Furthermore, other oxidative stress assays such as measurement of catalase and glutathione peroxidase could be chosen and incorporated in the study to obtain conclusive evidence on the effect of *D. hispida* extract on placental tissue.

## Figures and Tables

**Table 1:** Mass spectral characteristic of steroidal saponins determined by LCMS-ESI in the crude extract of *D. hispida*.

Peak No	Retention Time Rt (min)	Compound	Formula	Selected Ion	*m*/*z* Observed	Error (ppm)
i.	4.78	(+)Syringavesinol-4-*O*-β-d-glucopyranoside-isomer	C_28_H_35_O_13_	[M − H]^−^	579.20844	2.11
ii.	10.76	(+)Syringavesinol-4-*O*-β-d-glucopyranoside-isomer	C_28_H_35_O_13_	[M − H]^−^	579.20807	1.47
iii.	11.46	Dioscin	C_45_H_71_O_16_	[M − H]^−^	867.47941	6.62
iv.	12.06	Disogenin-3,6-dione	C_28_H_39_O_6_	[M − H]^−^	471.27484	1.54
v.	12.71	Progenin III	C_40_H_63_O_11_	[M − H]^−^	719.43701	0.72

## Sample Availability

Samples of the compounds are available from the authors.
